# Case Report: Multiple sclerosis and Neurofibromatosis type 1: a rare comorbidity

**DOI:** 10.3389/fneur.2026.1870007

**Published:** 2026-07-09

**Authors:** Angela Musci, Luigi Bonan, Eleonora Manzoni, Michele Carbonelli, Giulia Amore, Rocco Liguori, Valerio Carelli, Francesco Ventruto, Alessandra Lugaresi, Chiara La Morgia

**Affiliations:** 1 Department of Biomedical and NeuroMotor Sciences (DIBINEM), University of Bologna, Bologna, Italy; 2 IRCCS Istituto delle Scienze Neurologiche di Bologna, Bologna, Italy

**Keywords:** multiple sclerosis, neurofibromatosis type 1 (NF1), optic glioma, optic neuritis (ON), demyelinating lesions, focal area of signal intensity (FASI)

## Abstract

**Introduction:**

Neurofibromatosis type 1 (NF1) and Multiple Sclerosis (MS) are distinct neurological conditions with different underlying pathogenetic mechanisms. Their co-occurrence is exceedingly rare, with approximately 40 cases reported in the literature. Due to overlapping clinical and neuroimaging features, diagnosis is often challenging. We describe two cases of NF1-MS comorbidity highlighting the importance of a careful neuro-ophthalmological and neuroradiological evaluation for this rare association.

**Patient 1:**

A 33-year-old female presented with acute bilateral visual loss and bladder dysfunction. Brain and spinal cord Magnetic Resonance Imaging (MRI) showed multiple demyelinating lesions and a right globus pallidus Focal Area of Signal intensity (FASI); CSF exam showed oligoclonal bands, supporting a diagnosis of MS. Moreover, the identification of café-au-lait macules, optic pathway glioma (OPG) and Lisch nodules led to the discovery of a pathogenic NF1 variant.

**Patient 2:**

A 49-year-old male with left eye visual blurring, right optic nerve glioma, café-au-lait macules and subcutaneous neurofibromas was diagnosed with NF1. Initial brain and spine MRI identified a cervical neurofibroma and a dorsal intramedullary lesion, originally interpreted as a FASI. The patient developed new-onset genitourinary and sensory deficits. Follow-up brain MRI revealed new periventricular and subcortical demyelinating lesions, including a contrast enhancing one. Given its specific localization within the dorsolateral columns, the spinal lesion was redefined as a demyelinating plaque, supporting the diagnosis of definite MS.

**Conclusion:**

Our cases highlight two distinct diagnostic trajectories of NF1 and MS coexistence: MS as the presenting condition (Patient 1) and MS diagnosed during NF1 surveillance (Patient 2). The diagnostic challenge involves differentiating MS-related optic neuritis from OPG-associated visual symptoms and demyelinating plaques from FASIs. Furthermore, the management of MS co-occurring with NF1 is further complicated by the absence of established therapeutic guidelines. These cases highlight the importance of rigorous neuroimaging and neuro-ophthalmological evaluation in NF1 patients presenting new-onset symptoms or atypical MRI evolution.

## Introduction

Neurofibromatosis type 1 (NF1) is a multisystem autosomal dominant disorder resulting from pathogenic variants in the *NF1* gene. This gene encodes for neurofibromin, a tumour suppressor protein which acts as a negative regulator of the RAS proto-oncogene pathway. NF1 is mainly characterized by pigmentary lesions (café-au-lait macules, skinfold freckling and Lisch nodules), skeletal abnormalities and a predisposition to tumours of the skin and nervous system, including neurofibromas, optic pathway gliomas, and malignant peripheral nerve sheath tumours (1).

Multiple sclerosis (MS) is a chronic autoimmune neuroinflammatory disease that leads to demyelination and neurodegeneration of the central nervous system (CNS). Clinical features encompass visual disturbances, motor and sensory deficits, cerebellar dysfunction, and cognitive impairment (2).

The co-existence of neurofibromatosis type 1 (NF1) and multiple sclerosis (MS) is rare, with only a few cases reported in literature to date. This report describes two patients with this comorbidity, emphasizing the neuro-ophthalmological findings and the diagnostic challenge arising from shared clinical and radiological features.

## Case presentation

### Patient 1

A 33-year-old female presented with acute, painless bilateral visual loss. Her past medical history included an episode of left lower limb clumsiness occurring 1 year prior, followed by the development of urinary urgency and incomplete bladder emptying 6 months later. There was no family history of neurological disorders.

**Figure 1:**
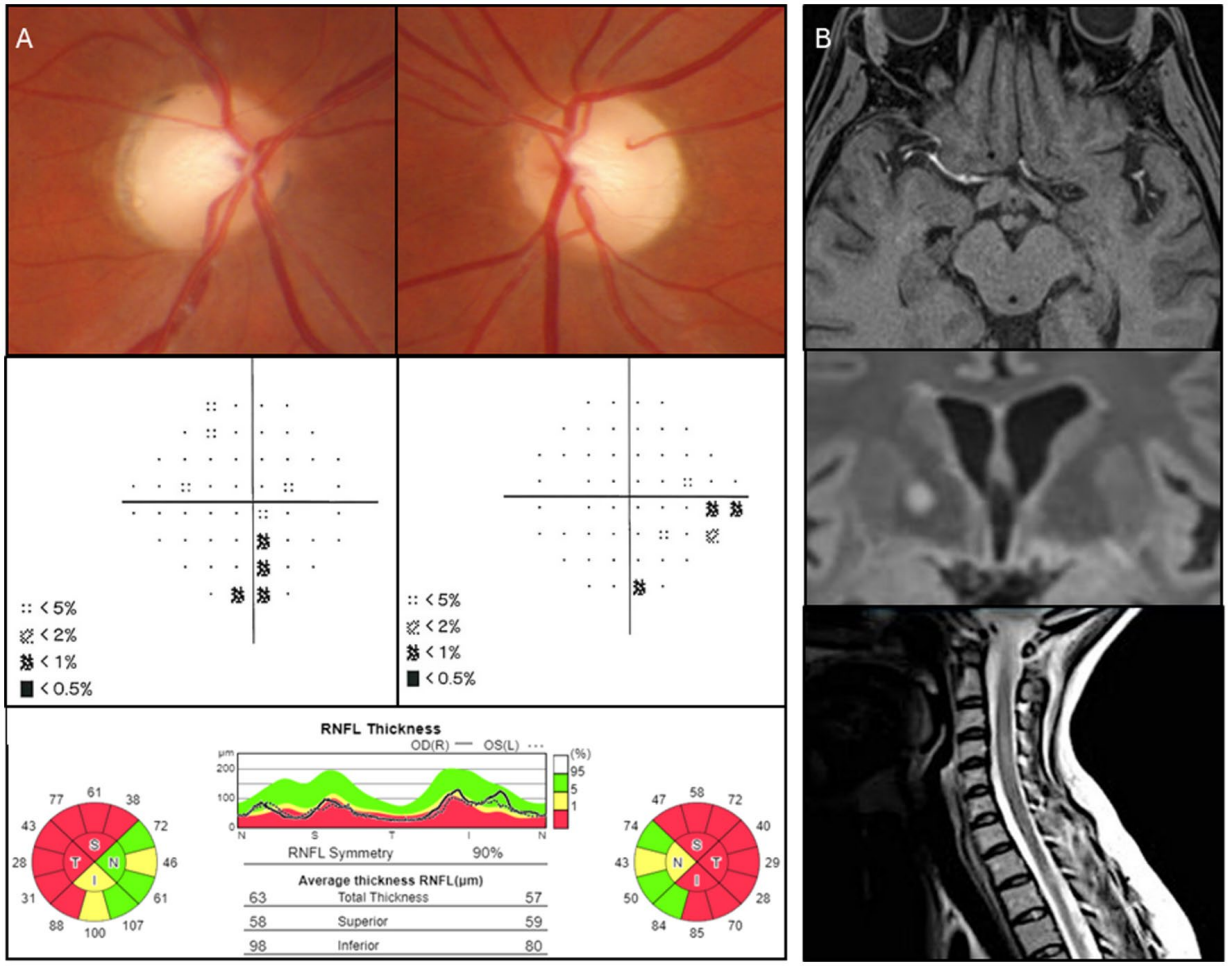
**(A)** Ophthalmological findings: in the upper panel fundoscopy shows bilateral optic disc pallor; in the middle panel visual fields (VF) show bilateral inferior defects; in the lower panel optical coherence tomography (OCT) shows bilateral thinning of the RNFL, with preservation of the nasal quadrant. **(B)** Radiological findings: in the upper panel axial T1-weighted Fat-Saturated brain MRI shows an optic glioma involving the right optic chiasma and right optic tract; in the middle panel coronal FLAIR T2-weighted brain MRI shows focal area of signal intensity (FASI) in the right globus pallidus; in the lower panel sagittal T2-weighted cervical spinal cord MRI shows multiple hyperintense demyelinating lesions.

Neurological examination showed mild left lower extremity weakness, hyperreflexia, bilateral sustained ankle clonus and extensor plantar responses. Ophthalmological assessment revealed a bilateral reduction in visual acuity (VA), expressed in LogMAR: right eye (RE) 0.5, left eye (LE) 0.8. Colour vision testing with Ishihara charts showed RE dyschromatopsia (RE: 1/12; LE 0/12). Fundoscopy identified bilateral optic disc pallor. Visual field (VF) testing demonstrated mild generalized threshold depression with inferior defects: RE mean defects (MD) -3.85 decibel (dB), LE MD -4.09 dB. Visual evoked potentials (VEPs) were not performed, while Optical Coherence Tomography (OCT) showed bilateral thinning of the Retinal Nerve Fiber Layer (RNFL), sparing the nasal quadrant (RNFL average thickness: RE 63 μm, LE 57 μm) (Figure 1A). Based on these findings, the patient was diagnosed with painless bilateral optic neuritis.

Brain and spinal cord magnetic resonance imaging (MRI) revealed multiple non-enhancing T2-weighted hyperintensities in the left optic nerve, periventricular regions, brainstem, cerebellar peduncles and cervical spine (C4-C5, C7) (Figure 1B). Cerebrospinal fluid (CSF) analysis revealed mild lymphocytic pleocytosis (11/mmc) and CSF-restricted oligoclonal bands, consistent with intrathecal immunoglobulin G (IgG) synthesis.

A diagnosis of relapsing–remitting MS (RRMS) was made according to the 2017 McDonald criteria. Alternative aetiologies were ruled out based on an extensive laboratory work-up, including fixed cell-based assay (CBA) negative testing for aquaporin-4-IgG (AQP4-IgG) and myelin-oligodendrocyte glycoprotein-IgG (MOG-IgG). She was treated with high-dose intravenous methylprednisolone (1 g daily for 5 days), leading to improvement in urinary and visual symptoms.

During hospitalization, the presence of cutaneous café-au-lait macules prompted further investigation. Re-evaluation of the brain MRI identified a T2-weighted hyperintensity in the right globus pallidus, exhibiting atypical distribution for MS, and an enhancing enlargement of the right chiasmal/optic tract, suggestive of an optic pathway glioma (OPG) (Figure 1B). Slit lamp examination showed bilateral Lisch nodules (LE > RE). Genetic testing confirmed a heterozygous pathogenic *NF1* variant (exon 33, c.3461 A > T; p. N1154I), establishing a diagnosis of NF1. Subsequently, the globus pallidus hyperintensity was interpreted as a Focal Area of Signal Intensity (FASI), based on its morphology and localization. We performed quantitative OCT and longitudinal MRI analyses. At one-year follow-up VA (+0.1 both eyes) and VF parameters had improved, while RNFL thickness remained stable (RNFL average thickness RE 57 μm, LE 54 μm). However, brain MRI revealed new punctiform periventricular demyelinating foci, including one gadolinium-enhancing. The patient was started on dimethyl fumarate, remaining clinically stable for 4 years. Subsequent ophthalmological examinations showed overall stability over 4 years (VA RE 0.63, LE 0.8, RNFL RE 53 μm, LE 52 μm).

However, by the third year of follow-up, brain and spinal cord MRI revealed an increased disease burden, including several contrast-enhancing lesions. Notably, the right chiasmal and optic tract glioma had grown, and the right globus pallidus hyperintensity had enlarged, developing a T2* hypointense rim. Despite radiological progression and considering the clinical stability, therapy was not escalated due to the immunosuppression and potential tumour progression risk associated with high-efficacy disease-modifying therapies (HEDMTs).

A five-year follow-up brain MRI showed further demyelinating lesions with contrast-enhancement, though the optic glioma and the globus pallidus FASI remained stable compared to the previous MRI. Given the inadequate MRI disease control with dimethyl fumarate and the low-risk JCV index of 0.32, treatment has been switched to natalizumab. This agent was selected among HEDMTs for its favourable risk–benefit profile regarding potential oncological progression risk, supplemented by a plan for future rigorous MRI surveillance.

### Patient 2

A 49-year-old man presented with recurring episodes of transient left eye visual blurring and headache triggered by physical exertion and lasting several hours. His medical history included benign prostatic hyperplasia and a family history of glaucoma.

**Figure 2:**
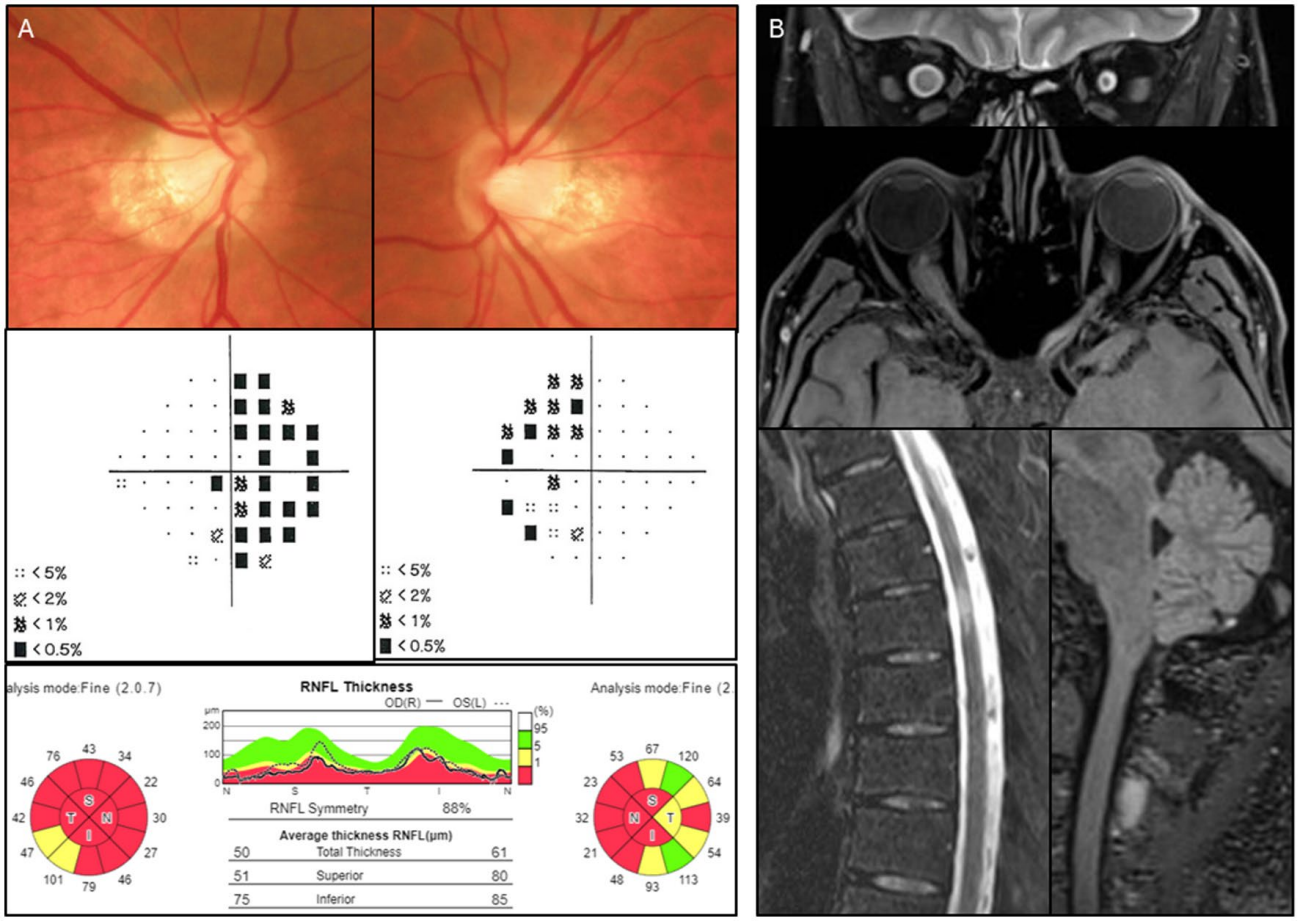
**(A)** Ophthalmological findings: in the upper panel fundoscopy shows small and tilted optic discs, with diffuse pallor of the right optic disc and temporal pallor of the left optic disc; in the middle panel VF show bitemporal hemianopia (OD > OS); in the lower panel OCT shows diffuse right generalized thinning of the RNFL and left optic bowtie atrophy. **(B)** Ophthalmological findings: in the upper panel fundoscopy shows small and tilted optic discs, with diffuse pallor of the right optic disc and temporal pallor of the left optic disc; in the middle panel VF show bitemporal hemianopia (OD > OS); in the lower panel OCT shows diffuse right generalized thinning of the RNFL and left optic bowtie atrophy. (B) Radiological findings: in the upper panel coronal T2-weighted brain MRI shows right optic nerve glioma and axial T1-weighted Fat-Saturated brain MRI shows chiasmal atrophy and right optic nerve glioma; in the lower panel sagittal STIR spinal cord MRI shows a demyelinating lesion in the dorsal spinal cord and sagittal FLAIR brain MRI shows a neurofibroma posterior to the C3 lamina.

Initial ophthalmological examination showed normal VA and normal fundoscopy. VF demonstrated bitemporal hemianopia (RE > LE) and an enlarged blind spot in the right eye (RE MD -5.55 dB, LE MD -2.24 dB) (Figure 2A). Brain computed tomography showed thickening of the right optic nerve. Subsequently, the patient consulted a neurologist and underwent a brain MRI which confirmed chiasmal atrophy and right optic nerve thickening with subtle sheath gadolinium enhancement, finding suggestive of optic nerve glioma (Figure 2B). Upon referral to our centre, fundoscopy revealed diffuse pallor of the right optic disc and temporal pallor of the left optic disc; both discs appeared small and tilted. VEPs were not assessed, while OCT showed right generalized thinning of the pRNFL (RNFL RE 50 μm, LE 61 μm) and left optic bowtie atrophy (Figure 2A).

Physical examination revealed numerous subcutaneous nodules on the trunk and extremities (histologically confirmed as neurofibromas), two café-au-lait macules and diffuse freckling. The diagnosis of NF1 was genetically confirmed by the identification of a heterozygous pathogenic *NF1* variant (exon 47, c.7037_7040delATAG, p. Asp2346ValfsTer49).

The baseline brain MRI also identified an ovoid, T1-hypointense, FLAIR-hyperintense enhancing lesion posterior to the C3 lamina; a dedicated spinal MRI later confirmed this finding as a neurofibroma. Additionally, a non-enhancing T2-hyperintense signal alteration was noted at the T7 level involving the dorsolateral columns, initially interpreted as a FASI (Figure 2B).

One year later, the patient reported new-onset bladder and sexual dysfunction. Neurological examination showed generalized hyperreflexia, dysreflexia (left > right), decreased vibratory sensation in the lower extremities and a mildly impaired tandem gait. In a follow-up brain scan, compared to the previous MRI, multiple T2-FLAIR hyperintense small areas within the periventricular and subcortical white matter were more easily detectable. Additionally, a further analogous signal alteration was described in the splenium of the corpus callosum. The distribution and morphology of these lesions strongly suggested an underlying demyelinating process.

A subsequent brain and spine MRI documented two new hyperintense areas in the frontal subcortical white matter and in the corpus callosum trunk, highly suggestive of MS. Following contrast administration, the callosal lesion showed pathological enhancement. The T7 lesion was redefined as a demyelinating area due to its dorsolateral location (Figure 2B). Despite the absence of CSF-restricted oligoclonal bands, a diagnosis of RRMS was established according to the 2017 McDonald criteria, after an extensive work-up ruled out alternative aetiologies (including negative CBA testing for AQP4-IgG and MOG-IgG).

Following the appearance of further periventricular lesions and one active enhancing lesion on a subsequent MRI, the patient started treatment with dimethyl fumarate. This led to an improvement in urinary symptoms and clinical stability. The patient underwent a longitudinal monitoring including sequential OCT and MRI analyses. Over a five-year follow-up, the patient has remained radiologically stable, with consistent neuro-ophthalmological parameters (RNFL average thickness: RE 47 μm, LE 62 μm).

## Discussion

We report two patients affected by the rare co-occurrence of NF1 and MS, characterized by different diagnostic trajectories. Patient 1 presented with optic neuritis (ON) and was diagnosed with both MS and NF1. According to the 2017 McDonald criteria, the MS diagnosis fulfilled the criteria for dissemination in space (DIS), confirmed by multiple MRI lesions in more than two CNS locations, and dissemination in time (DIT), established by a history of three clinical attacks and supported by CSF-restricted oligoclonal bands. The identification of multiple café-au-lait macules during physical examination raised suspicion of a concomitant condition. This was further corroborated by neuroimaging, which revealed a right OPG and T2-hyperintensities in the right globus pallidus, a location atypical for MS but highly suggestive of FASI. The diagnosis of NF1 was ultimately confirmed by the finding of Lisch nodules via slit-lamp examination and by genetic testing.

Consistent with the majority of cases described in the literature, MS diagnosis in Patient 2 was established during the longitudinal follow-up for NF1. This was triggered by new-onset genitourinary dysfunction, signs of spinal cord involvement and a new callosal lesion exhibiting characteristic demyelinating features. Follow-up neuroimaging identified an additional T2-hyperintensity involving the corpus callosum with active gadolinium enhancement, satisfying the 2017 McDonald criteria for dissemination in time (DIT). A critical diagnostic point was the longitudinal reassessment of the T2-hyperintense lesion at the T7 level. Initially interpreted as a FASI, its localization within the dorsolateral columns -a topographic distribution highly characteristic of MS- led to its reclassification as a demyelinating lesion. Notably, the occurrence of FASI within the spinal cord is exceedingly rare and sparsely documented in the literature. This spinal involvement, in conjunction with the periventricular lesions, fulfilled the 2017 McDonald criteria for dissemination in space (DIS), ultimately allowing a diagnosis of definite MS.

The co-occurrence of NF1 and MS is rare, with only 43 cases documented so far (3). While early reports on this association primarily described patients with primary progressive MS (PPMS) (4, 5), recent data show that RRMS and secondary progressive MS (SPMS) are also present in this population (6–8). Consistent with Patient 2, a minority of cases tested negative for CSF oligoclonal bands (3). Data regarding therapeutic management are limited. Earlier reports predominantly noted the use of interferon and immunosuppressants. Among presently used high-efficacy therapies, positive outcomes have been documented with natalizumab, fingolimod, dimethyl fumarate and with ocrelizumab (3). Neuro-ophthalmological and radiological features of the cases reported are summarized in Supplementary Table 1.

Epidemiological data from the French National Referral Centre for Neurofibromatosis revealed an increased tendency to develop MS in individuals carrying NF1 variants compared to the general population (8). Some hypotheses have been proposed regarding this co-existence.

Firstly, the greater incidence of MS among patients suffering from NF1 has been related to the presence of mutations in the oligodendrocyte myelin protein (*OMG*) gene, which is located within intron 27b of the *NF1* gene. The *OMG* gene encodes a membrane glycoprotein essential for CNS myelination and its defective function could lead to myelin structural anomalies, predisposing NF1 individuals to MS. One study analysing the *OMG* gene in four patients affected by both NF1 and PPMS found alterations in two individuals (5, 8). In Patient 1, *OMG* mutation analysis was negative, suggesting that *OMG* alterations alone cannot account for this comorbidity.

Another hypothesis involves the high expression of neurofibromin in oligodendrocytes, which play a direct role in MS-related neuroinflammation (9). Evidence from animal models suggests that *NF1* gene inactivation expands oligodendrocyte precursor cell populations and triggers nitric oxide-mediated blood–brain barrier disruption and myelin structural alterations (8, 10–12). Consequently, *NF1* loss-of-function may lead to significant oligodendrocyte dysfunction, promoting inflammation against CNS myelin.

Furthermore, neurofibromin inactivation leads to RAS pathway hyperactivation, promoting tumorigenesis. NF1 haploinsufficiency profoundly affects various immune cells, facilitating a hyper-inflammatory state (13). In NF1 patients, exposure to altered peripheral myelin might elicit an autoimmune response against CNS myelin. Further studies are required to elucidate this potential biological link.

The co-occurrence of MS and NF1 posits a significant diagnostic challenge due to the substantial overlap in clinical, ophthalmological and radiological features. Both conditions frequently involve the optic pathways. ON is the presenting symptom of MS in approximately 25% of patients and occurs in roughly 50% of patients over their disease course (14), while NF1 is associated with OPGs in 20% of cases (15). Notably, OPGs were identified in both our patients, whereas the first case also presented with ON. Both diseases cause visual impairment and similar ophthalmological signs, such as decreased VA, impaired colour vision, VF defects-including central scotomas. The clinical picture may progress to optic atrophy, detectable by peripapillary RNFL thinning and temporal pallor of the optic discs on fundoscopy (15, 16). Therefore, in case of vision loss, NF1 may mask concomitant demyelinating pathology, potentially delaying MS diagnosis and treatment.

Distinctive features that may suggest MS-related ON include subacute onset of visual decline, periocular pain exacerbated by extraocular muscle movements and profound dyschromatopsia, requiring recovery over weeks to months. Conversely, OPGs are characterized by chronic, painless and relentlessly progressive visual impairment. Thus, the acute onset of visual symptoms in patients with NF1 may point to MS-related ON. Radiologically, OPGs often appear hypo to iso-intense on T1 and hyperintense on T2 images, often showing uniform or patchy gadolinium enhancement; when localized strictly to the optic nerves they appear as well-defined, fusiform nerve enlargements, with severe kinking and tortuosity (15). In contrast, ON is characterized by a short-segment T2-hyperintensity of the optic nerve, with or without nerve sheath enhancement, reflecting focal inflammation rather than a proliferative process (16) (Table 1). While studies have demonstrated reduced average RNFL thickness in MS patients without optic neuritis due to chronic degeneration (14), it is noteworthy that a case of bilateral optic atrophy has also been described in an NF1 patient without evidence of OPGs (17). Furthermore, gait impairment, motor weakness and bladder or bowel dysfunction caused by MS-related spinal lesions may be erroneously ascribed to spinal root neurofibromas associated with NF1 (18).

**Table 1:** Differential diagnosis between MS-ON and NF1-OPGs.

Clinical and imaging feature	MS-ON	NF1-OPG
Temporal clinical onset	Acute or subacute (days to weeks)	Chronic, relentlessly progressive visual loss, frequently asymptomatic.
Associated pain	Typically painful, pain exacerbated by movement	Painless.
MRI Morphological Appearance	Focal, short-segment nerve involvement	Fusiform enlargement, severe nerve kinking, tortuosity, and elongation
MRI contrast enhancement	Strong, uniform gadolinium enhancement during the acute inflammatory phase	Highly variable enhancement (patchy, intensely uniform or absent)
OCT temporal dynamics (RNFL and mGCIPL)	Initial acute RNFL edema, followed rapidly by mGCIPL thinning, evolving to chronic RNFL atrophy	Chronic, progressive RNFL thinning correlating with tumor volume
Natural clinical history	Rapid onset followed by partial or complete spontaneous visual recovery over weeks or months.	Slow structural progression, prolonged stabilization or spontaneous tumor regression.

MS, multiple sclerosis; ON, optic neuritis; NF1, neurofibromatosis type 1; OPG, optic pathway glioma; MRI, magnetic resonance imaging; OCT, optic coherence tomography; RNFL, retinal nerve fiber layer; mGCIPL, macular ganglion cell-inner plexiform layer thickness.

Similarly, FASI often mimic demyelinating lesions, as both manifest on MRI as T2-weighted hyperintensities. However key radiological discriminators allow for a reliable differential diagnosis. FASI are typically iso- to hyperintense and non-enhancing on T1-weighted imaging; they are commonly located within deep gray matter and infratentorial structures, such as the basal ganglia, brainstem - especially the pons-, cerebellum, thalamus, and rarely involve cerebral hemispheres and spinal cord. In contrast, MS lesions frequently present as hypointensities on T1 images (“black holes”) and may exhibit gadolinium enhancement, a radiologic hallmark of active inflammation. They are typically located in the periventricular white matter, corpus callosum, cortical/juxtacortical areas, optic nerves and spinal cord. Finally, the natural history of these lesions differs significantly: MS is characterized by dynamic variations in size, number, and enhancement, whereas FASI usually increase in size and number until age 7 and regress by late adolescence (3, 19–21) (Table 2). Consequently, after ruling out malignancy, new adult-onset lesions in NF1 patients - as seen in Patient 2 - should not be automatically classified as FASI, but rather may suggest a demyelinating aetiology.

**Table 2:** Differential diagnosis between inflammatory demyelinating plaque and FASI.

MRI feature	Inflammatory demyelinating plaque	FASI
Primary topographical distribution	Periventricular white matter (Dawson’s fingers), corpus callosum, juxtacortical regions, and the spinal cord (specifically the dorsolateral columns).	Deep gray matter (basal ganglia, globus pallidus, thalamus), brainstem (pons), and cerebellar peduncles. Exceedingly rare in the spinal cord.
T1-weighted sequence characteristics	Isointense to severely hypointense; chronic destructive lesions evolve into permanent T1 “black holes.”	Isointense to mildly hyperintense; lacks destructive tissue necrosis.
T2-weighted and FLAIR sequences	Hyperintense.	Hyperintense.
Gadolinium contrast enhancement	Frequently present in active, acute inflammatory phases reflecting blood–brain barrier breakdown.	Absent.
Temporal evolution and natural history	Unpredictable dissemination in time; continuous emergence of new lesions and dynamic variations in size well into adulthood.	Emerge and proliferate in childhood, peak in early adolescence, and spontaneously regress or disappear completely by early adulthood.

MRI, magnetic resonance imaging; FASI, focal area of signal intensity.

Managing MS in NF1 patients is complex due to the lack of established therapeutic guidelines. Some studies have reported that prolonged exposure to DMTs could induce immunological response alterations and impair the host’s ability to perform immunosurveillance against malignant cells (22). Consequently, a rigorous individualized risk–benefit analysis is mandatory to balance MS-related disability prevention against the inherent predisposition of NF1 individuals to tumours (20). In Patient 1, given the highly active RRMS phenotype and the volumetric increase in both globus pallidus FASI and OPG throughout years, natalizumab was preferred as escalation therapy. Ocrelizumab and sphingosine-1-phosphate receptor modulators were avoided due to potential associations with an increased incidence of breast and skin malignancies (23). To date, only two published studies reported successful control of highly active MS with natalizumab in a patient with NF1 (20, 24). Further work is needed to establish standardized treatment protocols for this rare comorbidity.

## Conclusion

The neurological and radiological manifestations of MS can closely mimic NF1-related complications, often leading to diagnostic delays. Early diagnosis and timely intervention are crucial in MS to prevent further disability. Our cases emphasize that a meticulous neurological assessment, combined with a careful interpretation of longitudinal neuroimaging, is imperative for NF1 patients presenting with new-onset symptoms or atypical MRI evolution. Furthermore, therapeutic management remains critical, as standardized treatment guidelines for this specific comorbidity have yet to be established. Future research is needed to develop appropriate diagnostic and therapeutic strategies for this patient population.

## Data Availability

The original contributions presented in the study are included in the article/Supplementary material, further inquiries can be directed to the corresponding author.

## Author’s note

Prof. Carelli, Liguori, La Morgia are members of the European Reference Network for Neuromuscular Diseases – Project ID n°101156434.
