# Supplementary material for: Case Report: Multiple sclerosis and Neurofibromatosis type 1: a rare comorbidity
Source: Front Neurol. 2026 Jul 9;17:1870007. doi: 10.3389/fneur.2026.1870007 (PMC13391519; doi:10.3389/fneur.2026.1870007)
Supplement: Supplementary file 2 [file Table_1.docx]

Table 1: Key findings from the NF1-MS cases reported in the literature

| **Authors** | **Number of patients** | **Sex** | **Family history of MS** | **Family history of NF1** | **Genetics** | **Age at MS onset, years** | **MS pattern** | **CSF oligoclonal bands** | **Disease-modifying medications** | **Response to medication** |
| --- | --- | --- | --- | --- | --- | --- | --- | --- | --- | --- |
| Singh et al., 2025 | 1 | F | No | No | No data | 18 | RR | Yes | Ocrelizumab | Good response |
| Boukricha et al., 2024 | 1 | F | No | Yes (son) | No data | 42 | PP | Yes | No | / |
| Lemonaris et al., 2024 | 1 | F | No data | No data | Heterozygous de novo NF1 mutation (c.1527+4_1527+7delAGTA; NM_000267.3) | 27 | RR | Yes | First Interferon-β-1a, later Natalizumab | Inadequate disease control by Interferon-β-1a; good response to Natalizumab |
| Perucca et al., 2024 | 1 | F | No data | No data | No data | 29 | RR | Yes | Teriflunomide | No data |
| Kośmider et al., 2022 | 1 | F | No | Yes (both parents) | No data | 13 | RR | Yes | Dimethyl fumarate | Good response |
| Hadhiah et al., 2021 | 1 | F | No | No | Nonsense variant c.574C>T p.(Arg192*) in exon 5 of the NF1 gene | 22 | RR | Yes | First Interferon-β-1b, later Fingolimod | Inadequate disease control by Interferon-β-1b; good response to Fingolimod |
| Ciotti et al., 2021 | 1 | M | No | Yes | No data | 31 | RR | Yes | Natalizumab | Good response |
| Virgilio et al., 2021 | 1 | M | Yes | Yes | No data | 54 | PP | Yes | No data | No data |
| Carvalho et al., 2021 | 1 | M | No | Yes | Heterozygous mutation in the NF1 gene, c.888+1G>T | 24 | PP | Yes | Ocrelizumab | No data |
| Bergqvist et al., 2020 | 5 | No data | No data | No data | No data | 43(mean) | 3 RR, 2 SP | No data | No data | No data |
| Iwanowski et al., 2019 | 1 | F | No | No | De novo mutation c.6817delC and polymorphism rs1801052 in NF1, numerous polymorphisms in spastic paraplegia genes (SPG7, SPG15, SPG39) | 24 | PP | Yes | No data | No data |
| Gillani et al., 2017 | 1 | F | No data | No data | No data |  | SP | No | No data | No data |
| El-Heis et al., 2017 | 1 | M | No data | No data | No data | 33 | No data | Yes | No data | No data |
|  | 1 | F | No data | No data | No data | No data | No data | Yes | No | / |
|  | 1 | F | No data | No data | No data | 40 | No data | Yes | No | No response to oral methylprednisolone |
|  | 1 | M | No | No | No data | 30 | No data | Yes | No data | No data |
|  | 1 | F | No data | No data | No data | 30 | No data |  | No | No response to oral methylprednisolone |
| Solaf Elsayed et al., 2017 | 1 | F | No | Yes (son) | No data | 30 | RR | No | No data | Good response to high-dose steroid therapy |
| Mohebi et al., 2015 | 1 | F | No | Yes | No data | 32 | RR | Yes | No data | Good response to high-dose steroid therapy |
| Madubata et al., 2015 | 25 | No data | No data | No data | No data | No data | No data | No data | No data | No data |
| Pipatpajong et al., 2011 | 1 | M | No | No | No data | 27 | PP | No | No data | Mild and transient response to high-dose steroid therapy |
| Spinicci et al., 2010 | 1 | F | No | No | No data | 17 | RR | No | Immunomodulant drug | Good response to high-dose steroid therapy |
| Etemadifar et al., 2009 | 1 | F | No | Yes (mother) | No data | 19 | PP | No data | Interferon β-1a + Azathioprine | No data |
|  | 1 | F | Yes (sister) | Yes (father, two sisters) | No data | 33 | SP | Yes | Mitoxantrone | No data |
|  | 1 | F | Yes (sister) | Yes (father, two sisters) | No data | 36 | SP | No data | Methotrexate | No data |
|  | 1 | F | No | Yes (father) | No data | 30 | SP | No data | Interferon β-1b | No data |
|  | 1 | F | No | Yes (father) | No data | 26 | RR | No data | No | / |
|  | 1 | F | No | No | No data | 13 | RR | No data | Interferon β-1a | No data |
|  | 1 | M | No | Yes (mother) | No data | 16 | RR | No data | Interferon β-1b | No data |
| Perini et al., 2001 | 1 | M | No | No | No data | 19 | RR | Yes | No | Good response to high-dose steroid therapy |
|  | 1 | F | No | No | No data | 30 | SP | Yes | No | No response to high-dose steroid therapy in the progressive stage |
|  | 1 | M | No | Yes | No data | 46 | PP | Yes | No data | Mild and transient response to high-dose steroid therapy |
| Pal et al., 2001 | 1 | F | No data | No data | No data | 28 | P | Yes | Azathioprine | No response |
| Johnson et al., 2000 | 1 (+4 described by Ferner et al.) | M | No data | No data | No | 22 | PP | Yes | No data | No data |
| Ferner et al., 1995 | 1 | F | Yes (sister) | Yes (maternal grandmother, mother, sister) | No | 42 | PP | Yes | No | No response to high-dose steroid therapy |
|  | 1 | F | Yes (sister) | Yes (maternal grandmother, mother, sister) | No | 23 | PP | Yes | No | / |
|  | 1 | F | No | No | Glycine to aspartic acid polymorphic alteration at codon 21 | 26 | PP | Yes | No | No response to high-dose steroid therapy |
|  | 1 | F | No | No | No | 42 | PP | No | No | / |
|  | 1 | M | No | No | Glycine to aspartic acid polymorphic alteration at codon 21 | No data | PP | No data | No | No response to high-dose steroid therapy |

Table 2: Key findings from the NF1-MS cases reported in the literature

| **Authors** | **Number of patients** | **Sex** | **Eye symptoms** | **Neuro-ophthalmological signs** | **T2 weighted MRI** | **Gd enhancing lesions** |
| --- | --- | --- | --- | --- | --- | --- |
| Singh et al., 2025 | 1 | F | Bilateral blurry vision | Mild bilateral reduction of VA and normal fundoscopy | FASI in the basal ganglia and brainstem. Periventricular "Dawson's fingers" and juxtacortical demyelinating lesions. Demyelinating lesions in the cervical and dorsal spinal cord | Yes |
| Boukricha et al., 2024 | 1 | F | No | Normal examination | Demyelinating lesions in the periventricular regions, brainstem, and cerebellar peduncles | No |
| Lemonaris et al., 2024 | 1 | F | RE vision loss and periocular pain | RE Lisch nodules, right eye vision loss, dyschromatopsia and pale optic disc | FASI in the hippocampus and thalami. Demyelinating lesions in the right optic nerve and left internal capsule. Demyelinating lesions in the cervical and dorsal spinal cord | Yes |
| Perucca et al., 2024 | 1 | F | No | No data | Demyelinating lesions in the left thalamus and right pallidus. Demyelinating lesions in the cervical spinal cord.  Left optic nerve glioma | No |
| Kośmider et al., 2022 | 1 | F | Progressive vision loss | Progressive VA and VF impairment | FASI in deep brain structures and the midbrain. Bilateral optic nerve gliomas. Demyelinating lesions in the cervical spinal cord. | No |
| Hadhiah et al., 2021 | 1 | F | No | Normal examination | Demyelinating lesions in the centrum semiovale, periventricular regions, right pons and left cerebellar peduncle | No |
| Ciotti et al., 2021 | 1 | M | No | No data | Supra- and infra-tentorial demyelinating lesions. Demyelinating lesions in the cervical and dorsal spinal cord | Yes |
| Virgilio et al., 2021 | 1 | M | No | No data | Demyelinating WM and corpus callosum lesions. Demyelinating lesions in the cervical and dorsal spinal cord | No |
| Carvalho et al., 2021 | 1 | M | No | Bilateral reduction of VA, temporal pallor of the optic discs, RE central scotoma | FASI in the deep WM, the subcortical WM of the right frontal lobe, and both thalami. Demyelinating lesions in periventricular WM involving the corpus callosum. Demyelinating lesions in the spinal cord. | Yes |
| Bergqvist et al., 2020 | 5 | No data | No data | No data | No data | No data |
| Iwanowski et al., 2019 | 1 | F | No | Lisch nodules | Multiple brain and spinal cord lesions | No |
| Gillani et al., 2017 | 1 | F | No | No data | Periventricular and juxtacortical demyelinating lesions. Demyelinating lesions in the cervical and dorsal spinal cord | No |
| El-Heis et al., 2017 | 1 | M | No | No data | Demyelinating lesions in periventricular WM and spinal cord | Yes (neurofibroma) |
|  | 1 | F | No | No data | Brain and spinal cord demyelinating lesions | Yes (neurofibroma and schwannoma) |
|  | 1 | F | Visual cloudiness | Bilateral reduction of VA (RE 8/18, LE 5/6) and pale optic discs | Brain demyelinating lesions. Bilateral optic nerve gliomas | Yes |
|  | 1 | M | No | Right optic atrophy | Brain FASI. Demyelinating lesion in the cervical spinal cord | No |
|  | 1 | F | Recurrent attacks of vision loss |  | Periventricular demyelinating lesions. | No |
| Solaf Elsayed et al., 2017 | 1 | F | No | LE reduction of VA, normal fundoscopy | Demyelinating lesions in both periventricular regions, both centrum semiovale, right middle cerebellar peduncle and right side of midbrain. Demyelinating lesions in the cervical and dorsal spinal cord | Yes |
| Mohebi et al., 2015 | 1 | F | Binocular diplopia | Normal fundoscopy | Demyelinating lesions in periventricular and juxtacortical WM and in the cerebellum. Demyelinating lesion in the cervical spinal cord | Yes |
| Madubata et al., 2015 | 25 | No data | No data | No data | No data |  |
| Pipatpajong et al., 2011 | 1 | M | Double vision | / | Demyelinating lesions in periventricular regions ("Dawson's finger" pattern) and at right medial side of midbrain. Demyelinating lesions in the cervical and dorsal spinal cord | No |
| Spinicci et al., 2010 | 1 | F | LE visual blurring, double vision | Lisch nodules | Demyelinating lesions in periventricular WM (Dawson's fingers pattern), corpus callosum, subcortical regions and the brainstem. Demyelinating lesions in the cervical and dorsal spinal cord | Yes |
| Etemadifar et al., 2009 | 1 | F | Blurred vision,  Diplopia |  | Demyelinating lesions in the periventricular and juxtacortical regions | No |
|  | 1 | F | No | Right eye hamartoma | Demyelinating lesions in the periventricular and juxtacortical regions | No |
|  | 1 | F | No | No data | Demyelinating lesions in the periventricular and juxtacortical regions | No |
|  | 1 | F | Blurred vision | No data | Demyelinating lesions in the periventricular and juxtacortical regions. Demyelinating lesions in the cervical spinal cord | Yes |
|  | 1 | F | Blurred vision | No data | Demyelinating lesions in the periventricular and juxtacortical regions | Yes |
|  | 1 | F | Diplopia | / | Demyelinating lesions in the periventricular and juxtacortical regions and in the brainstem | Yes |
|  | 1 | M | No | No data | Demyelinating lesions in periventricular, juxtacortical and brainstem regions | Yes |
| Perini et al., 2001 | 1 | M | No data | No data | Demyelinating lesions in the centrum semiovale, periventricular regions and the corpus callosum | Yes |
|  | 1 | F | No | No data | Multiple brain and cervical spinal cord demyelinating lesions | No |
|  | 1 | M | No | No data | Demyelinating lesions in the periventricular regions, involving the corpus callosum | Yes |
| Pal et al., 2001 | 1 | F | No data | No data | Demyelinating lesions in the periventricular regions | No |
| Johnson et al., 2000 | 1 (+4 described by Ferner et al.) | M | No | Pale optic discs | Demyelinating lesions in the periventricular regions | No |
| Ferner et al., 1995 | 1 | F | No | Reduced VA (RE 6/18, RE 6/6-1), pale right optic disc | Demyelinating lesions in the centrum semiovale and periventricular regions | Yes |
|  | 1 | F | Bilateral impairment of colour vision | Bilateral optic atrophy | Demyelinating lesions in the periventricular regions, involving the corpus callosum | No data |
|  | 1 | F | No | Bilateral optic atrophy | Demyelinating lesions in the periventricular regions | No |
|  | 1 | F | No | No data | Demyelinating lesions in the periventricular region, pons, cerebellar hemispheres and lower medulla | No |
|  | 1 | M | No | Pale optic discs | Demyelinating lesions in the centrum semiovale, periventricular WM, cerebral peduncles and pons | No |

NF1, neurofibromatosis 1; MS, multiple sclerosis; CSF, cerebrospinal fluid; F, female; M, male; RR, relapsing remitting, PP, primary progressive; SP, secondary progressive; VA, visual acuity; FASI, focal area of signal alteration; RE, right eye; LE, left eye; VF, visual fields; WM, white matter.
